# Phosphoryl guanidine oligonucleotides as primers for RNA-dependent DNA synthesis using murine leukemia virus reverse transcriptase

**DOI:** 10.18699/VJGB-22-02

**Published:** 2022-02

**Authors:** E.S. Dyudeeva, I.A. Pyshnaya

**Affiliations:** Institute of Chemical Biology and Fundamental Medicine of the Siberian Branch of the Russian Academy of Sciences, Novosibirsk, Russia; Institute of Chemical Biology and Fundamental Medicine of the Siberian Branch of the Russian Academy of Sciences, Novosibirsk, Russia

**Keywords:** uncharged analogs of oligonucleotides, phosphoryl guanidine oligonucleotides, reverse transcription, reverse transcriptase, mouse leukemia virus reverse transcriptase, MMLV H-, незаряженные аналоги олигонуклеотидов, фосфорилгуанидиновые олигонуклеотиды, обратная транскрипция, ревертаза, обратная транскриптаза вируса лейкемии мышей, MMLV H-

## Abstract

Modern approaches to the detection and analysis of low-copy-number RNAs are often based on the use of RNA-dependent DNA polymerases, for example, in reverse-transcription PCR. The accuracy and eff iciency of cDNA synthesis in the reverse-transcription reaction catalyzed by reverse transcriptase (RNA-dependent DNA polymerase) signif icantly affect the correctness of the results of PCR diagnostic assays and/or RNA sequencing. In this regard, many studies are focused on the optimization of the reverse-transcription reaction, including the search for more perfect primers necessary to obtain a full-length DNA copy of RNA under study. The best-known completely uncharged analogs of oligonucleotides – morpholine oligonucleotides and peptide nucleic acids – cannot be substrates for enzymes that process nucleic acids. The aim of this work was to conduct a pilot study of uncharged phosphoryl guanidine oligodeoxyribonucleotides (PGOs) as primers for mouse leukemia virus reverse transcriptase (MMLV H-). Specif ic features of elongation of partially and completely uncharged PGO primers were investigated. It was demonstrated that PGOs can be elongated eff iciently, e. g., in the presence of a fragment of human ribosomal RNA having complex spatial structure. It was shown that the proportion (%) of abortive elongation products of a PGO primer depends on buffer ionic strength, nucleotide sequence of the primer, and the presence and location of phosphoryl guanidine groups in the primer. The results indicate the suitability of PGOs, including completely electroneutral ones, as primers for reverse-transcription PCR, thereby opening up new prospects for the creation of experimental models for the analysis of highly structured RNA.

## Abstract (ru)

Современные подходы к обнаружению и анализу низкокопийных РНК часто основываются на использовании РНК-зависимых ДНК-полимераз, например, метод ОТ-ПЦР. Точность и эффективность синтеза кДНК в реакции обратной транскрипции (ОТ), катализируемой ревертазой (РНК-зависимой ДНК-полимеразой), значительно влияют на корректность результатов ПЦР-диагностики и/или секвенирования РНК. В связи с этим множество исследований посвящено оптимизации реакции ОТ, в том числе поиску более совершенных праймеров, необходимых для наработки полноразмерной ДНК-копии исследуемой РНК. Наиболее известные полностью незаряженные аналоги олигонуклеотидов – морфолиновые олигонуклеотиды и пептидонуклеиновые кислоты – не могут являться субстратами для ферментов, процессирующих НК. Целью нашей работы было пилотное исследование незаряженных фосфорилгуанидиновых олигодезоксирибонуклеотидов (ФГО) в качестве праймеров для обратной транскриптазы вируса лейкемии мышей (MMLV H-). Изучены особенности превращения частично и полностью незаряженных ФГО-праймеров. Продемонстрировано, что ФГО способны эффективно удлиняться, в том числе в присутствии фрагмента рРНК человека, обладающего сложной пространственной структурой. Доля абортивных продуктов удлинения ФГО-праймера зависит от ионной силы использованного буферного раствора, нуклеотидной последовательности праймера и наличия и расположения в его составе ФГ-групп. Полученные данные показывают возможность использования ФГО, включая полностью электронейтральные, как праймеров в реакциях ОТ-ПЦР, что открывает новые перспективы для создания систем анализа высокоструктурированных РНК.

## Introduction

The detection of certain RNAs – some of the most important natural biopolymers – is an extremely relevant task in molecular diagnostics because such biomolecules are indicators of the state of the cell or an organism as a whole, of its metabolic status, or the presence of infection. Rapid and highly sensitive RNA detection can facilitate early diagnosis and hence more effective therapy for many diseases. Among RNA detection methods, the most popular is reverse-transcription polymerase chain reaction (RT-PCR), which consists of two sequential steps: (1) enzymatic synthesis of cDNA from an RNA template using reverse transcriptase and (2) obtaining the corresponding fragment of double-stranded DNA by standard PCR. With fluorophore-containing primers or probes, it is possible to carry out PCR in real time via monitoring an increase in the fluorescence signal, from which it is possible to accurately calculate the amount of analyzed RNA per cell/ sample. RT-PCR is widely employed in the research on mRNA expression patterns (Bustin, 2000), and this method is used at the stage of creation of libraries for high-throughput RNA sequencing (RNA-Seq): a powerful tool for investigation of transcriptomes and other purposes (Mortazavi et al., 2008; Haas, Zody, 2010).

A popular enzyme for the reverse-transcription reaction is the Moloney murine leukemia virus (MMLV) reverse transcriptase. This reverse transcriptase is capable of (a) catalyzing DNA synthesis from a DNA or RNA template (Palikša et al., 2018; Li R. et al., 2020); (b) template-free synthesis of short DNA fragments (Ohtsubo et al., 2017); (c) DNA synthesis with strand displacement (Kelleher, Champoux, 1998; Malik et al., 2017); (d) switching a template strand (Wulf et al., 2019); and (e) cleavage of RNA as part of a hybrid RNA–DNA complex (Schultz, Champoux, 2008; Li R. et al., 2020). In laboratory studies, a recombinant variant devoid of RNase H activity (MMLV H-) is used. An extensive body of research deals with optimizing the reverse-transcription reaction, including a search for more efficient primers based on modified oligonucleotides with enhanced affinity for complementary nucleic acids (Heuverswyn et al., 2016; Menéndez-Arias et al., 2016; Palikša et al., 2018; Li R. et al., 2020). The currently known completely uncharged analogs of oligonucleotides – morpholine oligonucleotides and peptide nucleic acids – cannot serve as a substrate of enzymatic reactions, probably owing to the unusual/ foreign backbone, which is substantially different in structure from the natural sugar-phosphate backbone. Partially modified P-alkyl phosphonate oligonucleotides and phosphoryl guanidine oligodeoxyribonucleotides (PGOs), in which the ribose moiety remains unchanged, are utilized as primers to distinguish the “wrong” complex during PCR (Li T.-L. et al., 2019; Chubarov et al., 2020). For PGOs, it has been demonstrated that they form complexes with complementary DNA or RNA in solutions with low ionic strength, and even in deionized water; thermal stability of these complexes is comparable to that of the native complex under conditions close to physiological (Kupryushkin et al., 2014; Dyudeeva et al., 2019). Moreover, it is reported that spatial structure of duplexes containing PGOs is virtually the same as the structure of the double helix of two native nucleic acids (Lomzov et al., 2019). The combination of these factors makes phosphoryl guanidine (PG)-containing oligonucleotides a promising platform for the development of highly specific probes and, possibly, enables their applications in some enzymatic transformations of nucleic acids catalyzed by either reverse transcriptase or DNA-dependent DNA polymerases (Kupryushkin et al., 2017; Chubarov et al., 2020).

The aim of this work was to study the effects of a number of PG groups and the profile of modification of a DNA primer with PG groups on the possibility of RNA-dependent elongation of such a primer by reverse transcriptase MMLV H- and to analyze the prospects of PGOs as primers for RT-PCR in the detection of relatively long RNAs.

## Materials and methods

Synthesis of nucleic acids. The synthesis of native oligodeoxyribonucleotides and their PG-containing analogs was carried out by the phosphoramidite method on an ASM-800 automated DNA/RNA synthesizer (Biosset, Russia) with commercial phosphoramidite monomers and porous polymer car-riers (Glen Research, USA). For the introduction of PG groups, the protocol described by Kupryushkin et al. (2014) was used. A fragment of human 18S ribosomal RNA (rRNA) was kindly provided by M.R. Kabilov (Institute of Chemical Biology and Fundamental Medicine of the Siberian Branch of the Russian Academy of Sciences).

The isolation of oligonucleotides and their PGO analogs from a reaction mixture was implemented by reversedphase high-performance liquid chromatography on an Agilent 1200 series chromatograph (Agilent, USA) with a column (4.6 × 150 mm) carrying the Eclipse XDB-C18 sorbent (5 μm) (Agilent). Elution of oligonucleotides was conducted in a linear concentration gradient of acetonitrile (0–90 %) in a 0.02 M solution of triethylammonium acetate for 30 min at a flow rate of 1.5 ml/min.

Electrophoretic analysis of oligonucleotides and their PGO analogs under denaturing conditions was performed in a 15 % polyacrylamide gel (PAAG; an acrylamide:N,N′- methylene bisacrylamide ratio of 29:1, with 8 M urea) in TBE buffer (89 mM Tris-borate, pH 8.3, 2 mM Na2EDTA) at a voltage of 50 V/cm. The results of electrophoretic separation were visualized by staining the gel with the StainsAll reagent. When a fluorophore was present in oligonucleotides, fluorescence scanning was performed by means of a VersaDoc MP 4000 Molecular Imager System (Bio-Rad, USA), which is a gel documentation system.

To separate relatively long products of primer elongation obtained by a reverse-transcription reaction, we used a 1.5 % agarose gel in TBE buffer in a horizontal electrophoretic unit (Bio-Rad). Double-stranded-DNA molecular-weight markers called the GeneRuler 100 bp DNA Ladder (ThermoScientific, Lithuania) were employed to assess relative electrophoretic mobility of the samples. The results of electrophoretic separation were visualized by staining the gel with ethidium bromide (0.0001 % aqueous solution) and by scanning the fluorescence signal as described above. The proportion (%) of major primer elongation products was calculated by analyzing the scanned fluorescence signal of the respective spots by means of the GelPro Analyzer 4.0 software (Media Cybernetics, USA).

Reverse-transcription reactions. Enzymatic elongation of oligonucleotides and their PGO analogs in a reverse-transcription reaction was performed using a recombinant enzyme: Murine leukemia virus reverse transcriptase MMLV H- (SibEnzyme, Russia). The following solutions served as buffers: standard (St): 30 mM Tris-HCl, 5 mM MgCl2, 50 mM KCl, 5 mM dithiothreitol, and low-salt (L): 30 mM Tris-HCl, 2 mM MgCl2, 5 mM dithiothreitol. Amounts of components of the reverse-transcription reaction mixtures are presented and compared in Table 1 (unless indicated otherwise); the total volume of the mixture was 10 μl.

**Table 1.**
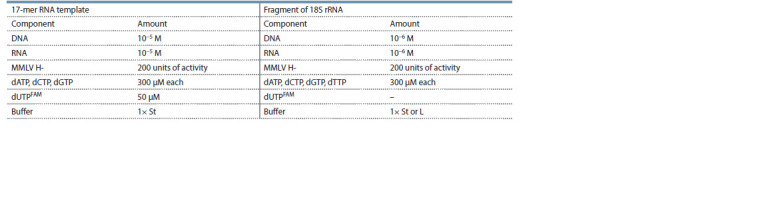
Composition of reaction mixtures for reverse transcription in the case of a short synthetic RNA template and a relatively long fragment of rRNA

The reverse-transcription reaction was conducted at 37 °C. The reaction time was 1 h in the case of the 17-mer RNA template and 3 h for a high-molecular-weight RNA template: a fragment of human 18S rRNA. At the end of the reaction, the enzyme was thermally inactivated by keeping the reaction mixture at 95 °C for 5 min, or the RNA template was hydrolyzed by adding an excess of concentrated aqueous ammonia to the reaction mixture and incubating at 56 °C for 2 h. Ammonia was removed by evaporating the solutions in a vacuum concentrator until the odor disappeared.

## Results and discussion

**Fig. 1.**
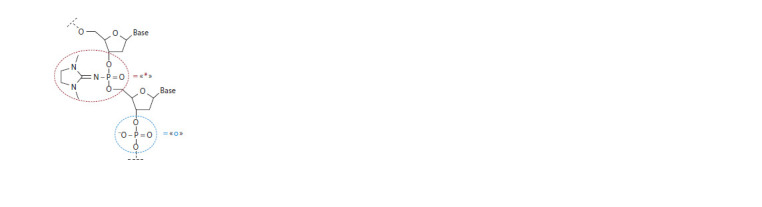
Structures of the modif ied phosphate group (i. e., the PG group, indicated by the asterisk) and native phosphate (indicated as “o”) in a DNA strand. Base: a nitrogenous base.

At the first stage of the work, we examined a series of decameric deoxyribonucleotides identical in nucleotide sequence but differing in the number of uncharged groups (i. e., PG groups) that were introduced (Fig. 1) and in their mutual arrangement (Table 2).

**Table 2.**
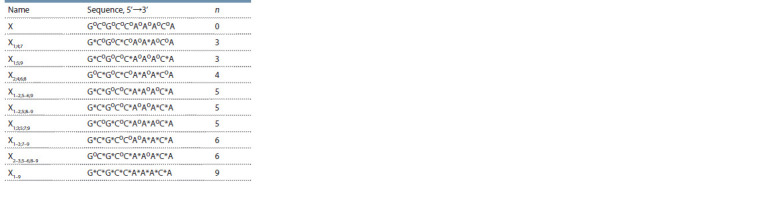
Structures of decanucleotides Notе. “o”: a native internucleotide phosphate group, n: the number of PG groups, which are denoted by “* ”. In the proposed names, “ X” is the general name of the series of 10-mer oligonucleotides, and the subscript designates position numbers (counted from the 5’ end) of the PG groups. All decanucleotides were isolated and purified in a uniform manner (see the Materials and methods section).

**Fig. 2.**
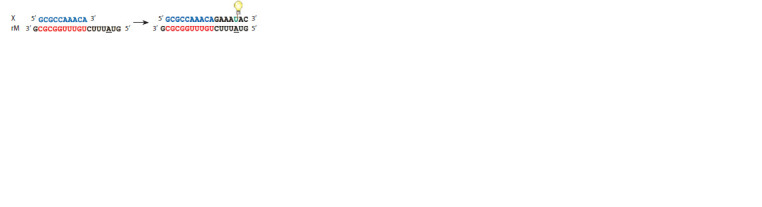
The scheme of elongation of an X series decanucleotide along the rM RNA template with the formation of a f luorescent product.

The analyzed decanucleotides were complementary to the 3′-terminal region of a model 18-mer RNA template called rM (Fig. 2). The nucleotide sequence of the template as part of the formed complex contained only one rA position in the single-stranded part, opposite to which it was possible to insert only one fluorescent dUMPFAM residue into the relatively long DNA chain using the DNA-dependent RNA polymerase under study: Moloney murine leukemia virus reverse transcriptase (MMLV H-).

**Fig. 3.**
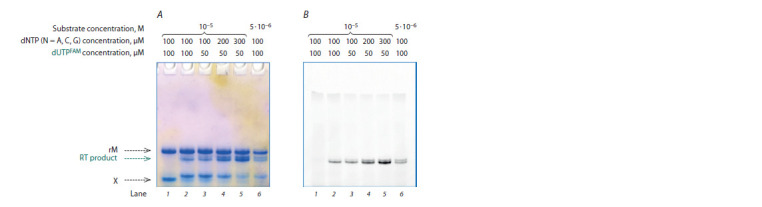
Electrophoretic separation of the products of reverse-transcription reactions involving native substrate X–rM at 37 °C and 200 U of the enzyme at various concentrations of triphosphates and the substrate (i. e., a DNA–RNA complex, as shown in the f igure) in a denaturing 15 % PAAG after visualization with the StainsAll reagent (A) and scanning of the f luorescence signal (B). Arrows indicate the template (rM), a reverse-transcription reaction product, and the native decanucleotide (X).

Initially, optimal conditions of the reverse-transcription reaction were found using a native substrate (the X–rM complex) as an example. A series of experiments was conducted in which we varied (1) the reaction temperature, (2) the amount of the enzyme, or the concentration of (3) triphosphates or (4) the substrate DNA–RNA complex. The products of the reverse-transcription reaction were separated in a 15 % PAAG and visualized through scanning the fluorescence signal in the FAM (6-carboxyfluorescein) channel and then staining the same gel with the StainsAll reagent. It should be noted that staining the gel with StainsAll allows to examine the material balance of each reaction because this dye “reveals” all elongation products as well as the original template and primer. The fluorescent signal in turn only reflects the accumulation of the target products of the reverse-transcription reaction, with a length of at least 15 nucleotides, i. e., products containing the incorporated dUMPFAM residue. Figure 3 illustrates a typical result of a reverse-transcription reaction analysis.

It is obvious that an increase in the concentration of fluorescent dUTPFAM, with all other things being equal (see Fig. 3, lanes 2 and 3), does not affect accumulation efficiency of elongation products of the native primer in the X–rM complex. On the contrary, when the concentration of unlabeled dNTP was increased at a constant concentration of dUTPFAM (see Fig. 3, A, lanes 3–5), a decrease in the proportion of abortive products of primer elongation was observed and, as a consequence, a higher yield of the fluorescent elongation product. The highest yield of the full-size reaction product (according to the fluorescence signal in Fig. 3, B) with the lowest amount of abortive elongation products is seen in lane 5.

**Fig. 4.**
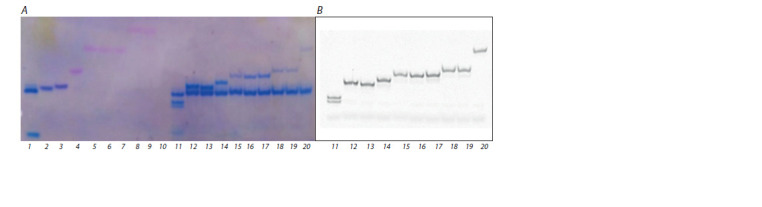
Electrophoretic separation of the products of reverse-transcription reactions in a denaturing 15 % PAAG after staining with the StainsAll reagent (A) and scanning the f luorescence signal (B). Lanes 1–10 represent controls without the enzyme: mixtures of rM and primer X (lane 1) or primer X1;4;7 (lane 2), X1;5;9 (lane 3), X2;4;6;8 (lane 4), X1–2;5;8–9 (lane 5), X1;3;5;7;9 (lane 6), X1–2;5–6;9 (lane 7 ), X1–3;7–9 (lane 8), X2–3;5–6;8–9 (lane 9), X1–9 (lane 10); lanes 11–20 represent reverse-transcription reactions involving primer X (lane 11), X1;4;7 (lane 12), X1;5;9 (lane 13), X2;4;6;8 (lane 14), X1–2;5;8–9 (lane 15), X1;3;5;7;9 (lane 16), X1–2;5–6;9 (lane 17 ), X1–3;7–9 (lane 18), X2–3;5–6;8–9 (lane 19), X1–9 (lane 20).

It is these conditions that were chosen subsequently to compare the efficiency of the reverse-transcription reaction of native primers versus that of PG-containing primers, the backbone of which partially or completely lacked the negative charge. Figure 4 presents data from the electrophoretic analysis of the products of the reverse-transcription reaction involving complexes of rM with each decanucleotide as a substrate (the decanucleotides are described in Table 2).

According to both the results on the gel stained with StainsAll (see Fig. 4, A) and the fluorescence signal data (see Fig. 4, B), it can be argued that there was an enzymatic elongation of all the oligonucleotides, including completely electroneutral X1–9. It should be pointed out that the accumulation of a full-size fluorescent product slightly decreased with an increase in the number of PG groups in a primer (see Fig. 4, B, lanes 11–20). This result is probably due to disturbances of electrostatic interactions within the enzyme–substrate complex. Nevertheless, our data indicate that uncharged PGOs can serve as primers in a reverse-transcription reaction; to the best of our knowledge, this finding has never been reported regarding completely modified uncharged oligonucleotide analogs.

It was not possible to analyze in detail the degree of elongation of PGO primers in this model system (see Fig. 4, A, lanes 2–10 and 11–20) because a higher degree of modification of the oligos sharply reduces the efficiency of their staining with dyes specific to nucleic acids, for example, StainsAll (Dyudeeva et al., 2019). Taking this circumstance into account, native and PG-containing oligonucleotides carrying a fluorophore at the 5′ end were designed for the next stage of the work (Table 3). These materials enabled subsequent detection of the entire spectrum of products of enzymatic elongation of primers on a relatively long template: a fragment of human 18S rRNA. The primer structures varied in the degree of modification of their sugar-phosphate backbone at the 3′ end. Along with native primers (J1.o and J2.o), we used their partially uncharged analogs containing a backbone modified at the 5′ end and unmodified at the 3′ end with six unmodified phosphate groups (J1.6 and J2.6) or three unmodified phosphate groups (J1.3 and J2.3) as well as completely electroneutral analogs (J1.x and J2.x, fully modified backbone).

**Table 3.**
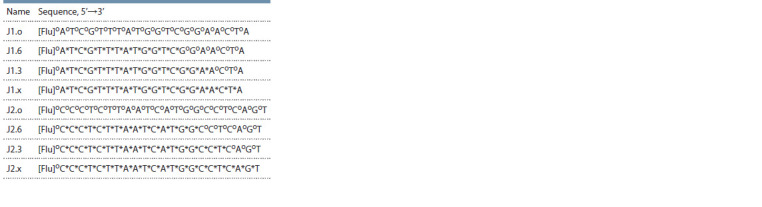
Structures of primers for the reverse-transcription reaction involving a high-molecular-weight RNA template: a fragment of human 18S rRNA Notе. “o”: a native internucleotide phosphate group, “*”: a PG group, [Flu]: a f luorescein residue.

**Fig. 5.**
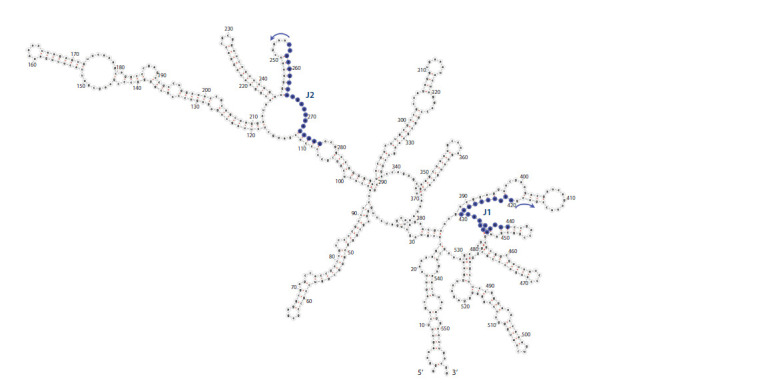
Hypothetical secondary structure of the fragment of human 18S rRNA, as built using an online service, RNAfold web server. J1 and J2: sites selected for primer hybridization. Arrows indicate the direction of primer elongation.

The nucleotide sequences of these primers were complementary to some regions of the fragment of human 18S rRNA (the length of the fragment was 558 nucleotides) (Fig. 5).

The first of the two sites selected for hybridization – the J1 series in Fig. 5 – is located closer to the 3′ end region of the fragment of rRNA. Through reverse transcription from this site, it is possible to obtain products, the theoretical length of which is 439 nucleotides. The second binding site of the J2 series primers is located closer to the middle of the rRNA fragment; theoretical size of the full-length elongation products of these primers is 276 nucleotides. When choosing binding sites for oligonucleotides in structured RNA templates, we employed the principles laid down by Petyuk et al. (1999).

**Fig. 6.**
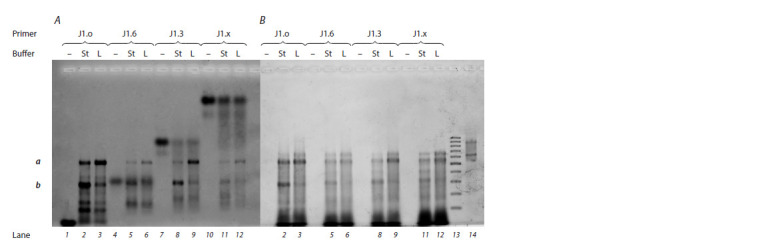
Data on the scanning of f luorescence signals of FAM (A) or ethidium bromide (B) after separation of the reversetranscriptional reaction products in a 1.5 % agarose gel: individual DNA primers J1.o (lane 1), J1.6 (lane 4), J1.3 (lane 7 ), and J1.x (lane 10), which are visible only when FAM f luorescence is scanned; products of the reverse-transcription reaction involving primer J1.o (lane 2), J1.6 (lane 5), J1.3 (lane 8), or J1.x (lane 11) under “standard” buffering conditions (St); products of the reverse-transcription reaction involving primer J1.o (lane 3), J1.6 (lane 6), J1.3 (lane 9), or J1.x (lane 12) in the buffer with low ionic strength (L); DNA molecular-weight markers 100–1000 bp (lane 13), which are visible only when the f luorescence of ethidium bromide is scanned; the RNA template (lane 14), which is visible only when the f luorescence of ethidium bromide is scanned. The types of primer and buffer are indicated in the f igure; a and b correspond to positions of the prevalent products of the reverse-transcription reaction

PGOs, as previously demonstrated, can form a complementary complex in solutions with ultralow ionic strength (Kupryushkin et al., 2014; Dyudeeva et al., 2019); therefore, it was essential to compare the efficiency rates of the reversetranscription reactions involving native versus PG-containing primers in buffers with different ionic strengths. For this purpose, potassium chloride was completely removed from the “standard” buffer, and the concentration of magnesium chloride was reduced from 5 to 2 mM. A decrease in ionic strength of the buffer may help to destabilize internal secondary structure of an RNA template, thereby “facilitating” the reverse-transcription reaction. Relatively long products of the reverse-transcription reaction were analyzed by electrophoresis in a 1.5 % agarose gel, with detection of the primer elongation products by means of the fluorescence signal and by staining with ethidium bromide. The results of electrophoretic separation of reverse-transcription reaction products obtained in buffers with “standard” and “low” ionic strength for primers of the J1 series are given in Fig. 6. Readers can see that the introduction of PG groups into the primer leads to an expected decrease in its electrophoretic mobility owing to a decrease in the total charge of the molecule (see Fig. 6, A, lanes 1, 4, 7, and 10). Enzymatic elongation of a primer was observed in all cases, even for primer J1.x, which has a completely electroneutral sugar-phosphate backbone (see Fig. 6, A, lanes 11 and 12).

Electrophoretic mobility of product a is comparable to that of the initial RNA template (see Fig. 6, B, lane 14), which is 558 nucleotides long, and to the mobility of 500 and 600 bp bands among the DNA molecular-weight markers (lane 13). It can be assumed that this product is close to the theoretical full-length one. Product b has lower electrophoretic mobility approximately corresponding to 300 bp among the doublestranded- DNA molecular-weight markers. Even the introduction of an additional step of denaturation of rRNA at 95 °C to reduce the likelihood of structural complexity did not raise the yield of product a (data not shown).

Notably, electrophoretic mobility of initial primers J1.6, J1.3, and J1.x (see Fig. 6, A, lanes 4, 7, and 10) is lower than that of the products of their elongation by the reversetranscription reaction (lanes 5–6, 8–9, and 11–12). Apparently, at initial stages of the reaction, the inclusion of dNTPs into the growing chain of the PGO primer causes an increase in the migration rate of the oligonucleotide in the gel owing to an appreciable change in the charge; the size of the molecule has not yet changed so much as to cause product retardation in agarose gel pores. Nevertheless, when a certain critical length is reached at later stages of the reaction, the total molecular size has a greater effect on the migration rate in the gel than does the increase in charge when each subsequent dNTP is incorporated. Thus, relatively long products of the reverse-transcription reaction involving PG-containing primers manifested “classic” behavior in the gel, by slowing down with increasing molecular size. In this context, the influence of the relatively small number of PG groups becomes negligible. For this reason, it is likely that electrophoretic mobility of the major products of the reverse-transcription reaction (a and b) is almost independent from the number of modifications in the initial PG-containing primer.

A decrease in ionic strength of reaction buffer (see Fig. 6, lanes 3, 6, 9, and 12) leads to the accumulation of longer products in the reverse-transcription reaction as compared to standard reaction conditions (lanes 2, 5, 8, and 11) regardless of the presence of modifications in the primer. Ionic strength caused the most pronounced change in the ratio of elongation products for native primer J1.o: according to fluorescence intensity levels, the proportion (%) of the longest product a increased threefold (from 9 to 27 %), while the proportion of abortive product b diminished (from 26 up to 10 %) under the influence of the decrease in buffer ionic strength. Perhaps the lower ionic strength of the buffer destabilized the internal structure of RNA but did not have a significant impact on the formation of the DNA–RNA–enzyme triple complex. For PGcontaining primers, the redistribution of elongation products upon buffer change was observed too but at a level of only several percentage points.

It should be mentioned that for completely electroneutral PGO J1.x (see Fig. 6, А, lanes 10–12), we noticed less active elongation in the reverse-transcription reaction in comparison with the native primer (lanes 1–3). It is possible that the rate-limiting stage of the reverse-transcription reaction for the completely electroneutral PGO is the attachment of the first dNMPs to its 3′ end (consistent with the findings of Skasko et al., 2005). After that, the enzyme focuses on the “preferred” substrate (which contains native unmodified nucleotides at the 3′ end) and “ignores” even an excessive amount of the original primer.

**Fig. 7.**
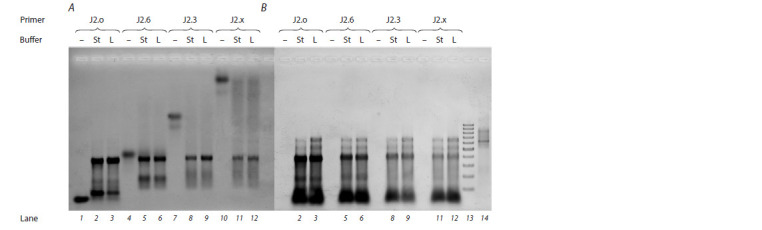
Data on the scanning of the f luorescence signal of FAM (A) or ethidium bromide (B) after separation of the reverse-transcription reaction products in a 1.5 % agarose gel: for individual DNA primers J2.o (lane 1), J2.6 (lane 4), J2.3 (lane 7 ), and J2.x (lane 10), which are visible only when FAM f luorescence is scanned; for products of the reverse-transcription reaction of primer J2.o (lane 2), J2.6 (lane 5), J2.3 (lane 8), or J2.x (lane 11) under the “standard” buffering conditions (St); for products of the reverse-transcription reaction of primer J2.o (lane 3), J2.6 (lane 6), J2.3 (lane 9), or J2.x (lane 12) in the buffer with low ionic strength (L); DNA molecular-weight markers 100–1000 bp (lane 13), which are visible only when ethidium bromide f luorescence is scanned; the RNA template (lane 14), which is visible only when the f luorescence of ethidium bromide is scanned. The types of primer and buffer are indicated in the f igure.

Similar results were obtained for primers of the J2 series (Fig. 7). In this case, fewer abortive products are observed among the primer elongation products. Most likely, the reason for this is that the theoretical maximal length of elongation products is shorter than that in the case of primer J1, and therefore during elongation, fewer hairpins or other structural complexities of the RNA template have to be overcome, and this situation makes the enzyme more processive.

**Fig. 8.**
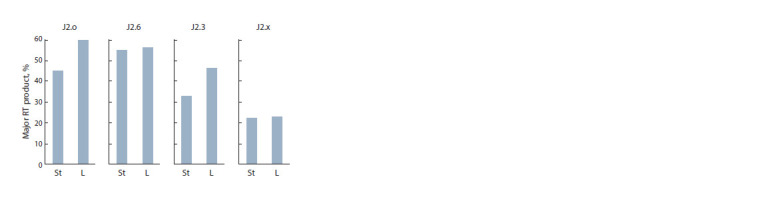
The proportion of the main product of elongation for primers of the J2 series in the reverse-transcription reaction carried out in a solution with “standard” (St) and low (L) ionic strength.

The main elongation product of the J2 series primers has an electrophoretic mobility comparable to that of a 400 bp double-stranded-DNA molecular-weight marker. Presumably, this product has a size similar to the theoretical full length (276 nucleotides for this primer). Its proportion (%) in the reaction mixtures is depicted in Fig. 8. As was the case for primers of the J1 series, we noticed greater accumulation of the target product in the buffer with lower ionic strength (L) for native primer J2.o (an increase in the proportion of the main product from 45 to 61 %) and for PGO primer J2.3 (an increase from 33 to 46 %, respectively).

Notably, other PGO primers turned out to be virtually indifferent to the changes in ionic strength. It is conceivable that the arrangement of PG groups in the active center of the enzyme affects the conformation of the enzyme–substrate complex and therefore its sensitivity to ionic composition of the medium and to solvation.

## Conclusion

Thus, we characterized the elongation of partially and completely uncharged PGOs in the reverse-transcription reaction carried out by MMLV H-. It was demonstrated that PG-containing primers can be elongated effectively, e. g., in the pre- sence of a fragment of human rRNA with complicated spatial structure. A decrease in buffer ionic strength raises the efficiency of full-length cDNA synthesis when either a native or PG-containing primer is used. The findings require further research and point to the suitability of PGOs, including completely electroneutral ones, as primers for RT-PCR, thereby opening up new prospects for the creation of experimental models for the analysis of highly structured RNA.

## Conflict of interest

The authors declare no conflict of interest.
